# Supplementary material for: High-Quality Predicted Pathway Annotations Greatly Improve Pathway Enrichment Analysis of Metabolomics Datasets
Source: bioRxiv. 2025 Nov 19:2025.11.18.689105. Preprint. [Version 1] doi: 10.1101/2025.11.18.689105 (PMC12667988; doi:10.1101/2025.11.18.689105)
Supplement: Supplement 1 [file NIHPP2025.11.18.689105v1-supplement-1.pdf]

bioRxiv preprint doi: https://doi.org/10.1101/2025.11.18.689105; this version posted November 19, 2025. The copyright holder for this preprint (which was not certified by peer review) is the author/funder, who has granted bioRxiv a license to display the preprint in perpetuity. It is made available under aCC-BY 4.0 International license.

Supplemental Material for

High-Quality Predicted Pathway Annotations Greatly Improve Pathway Enrichment Analysis of Metabolomics Datasets

Erik D. Huckvale (EDH)[1], P. Travis Thompson (PTT)[1], Robert M. Flight (RMF)[1], and Hunter N.B. Moseley (HNBM)[1,2,3,4,*]

[1]Markey Cancer Center, University of Kentucky, Lexington, KY, 40506, USA.

[2]Superfund Research Center, University of Kentucky, Lexington, KY, 40506, USA.

[3]Department of Molecular and Cellular Biochemistry, University of Kentucky, Lexington, KY, 40506, USA.

[4]Institute for Biomedical Informatics, University of Kentucky, Lexington, KY, 40506, USA.

*Corresponding author

EDH erik.huckvale@uky.edu

PTT ptth@g.uky.edu

RMF rflight79@gmail.com

HNBM hunter.moseley@uky.edu

bioRxiv preprint doi: https://doi.org/10.1101/2025.11.18.689105; this version posted November 19, 2025. The copyright holder for this preprint (which was not certified by peer review) is the author/funder, who has granted bioRxiv a license to display the preprint in perpetuity. It is made available under aCC-BY 4.0 International license.

Filtering Counts

Table 1 - The number of MW datasets filtered by reason for filtering.

**Reason to Filter**	**#Datasets Filtered**
Neither KEGG nor PubChem compound ID field	2,356
Insufficient number of metabolites	900
Metabolite intensities on the wrong scale	113
Software failure	8
Unreadable file	5

Pre-GSEA

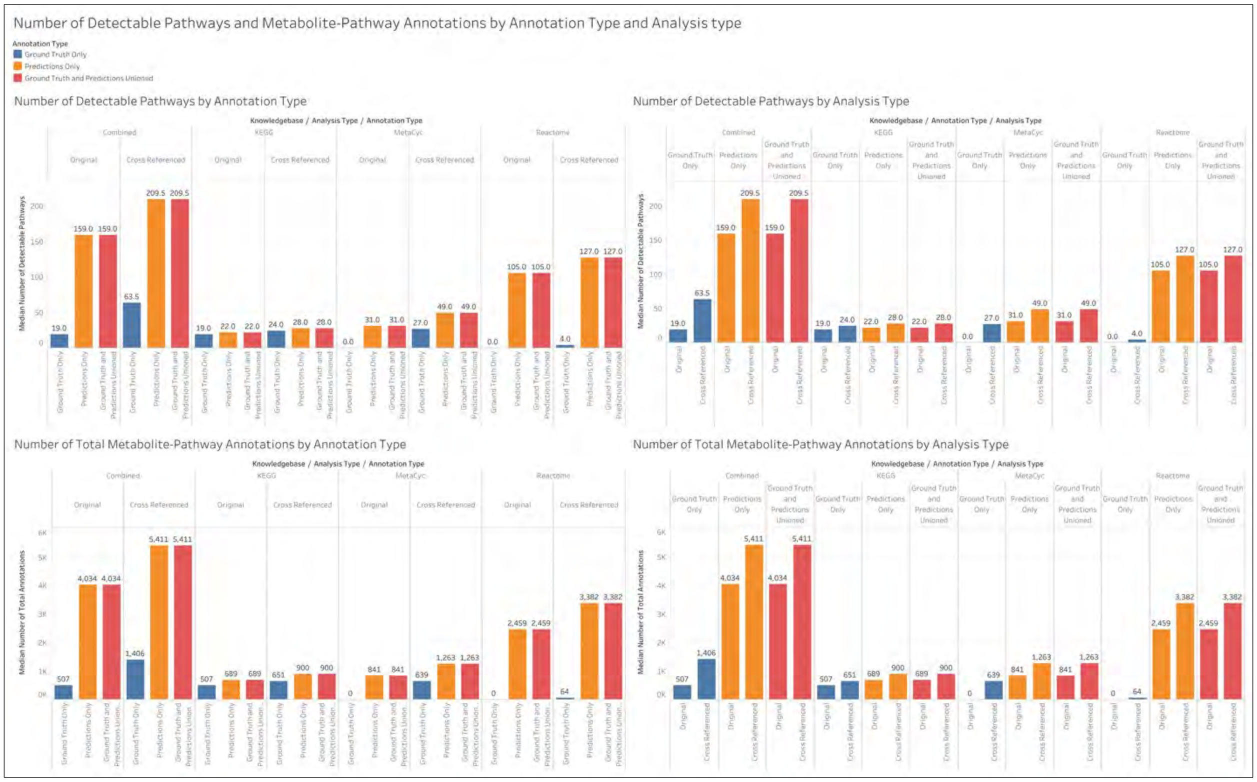

Figure 1 - Comparing the median number of detectable pathways and tot metabolite-pathway annotations between original and cross-referenced MW datasets and between ground truth and predictions for each knowledgebase.

bioRxiv preprint doi: https://doi.org/10.1101/2025.11.18.689105; this version posted November 19, 2025. The copyright holder for this preprint (which was not certified by peer review) is the author/funder, who has granted bioRxiv a license to display the preprint in perpetuity. It is made available under aCC-BY 4.0 International license.

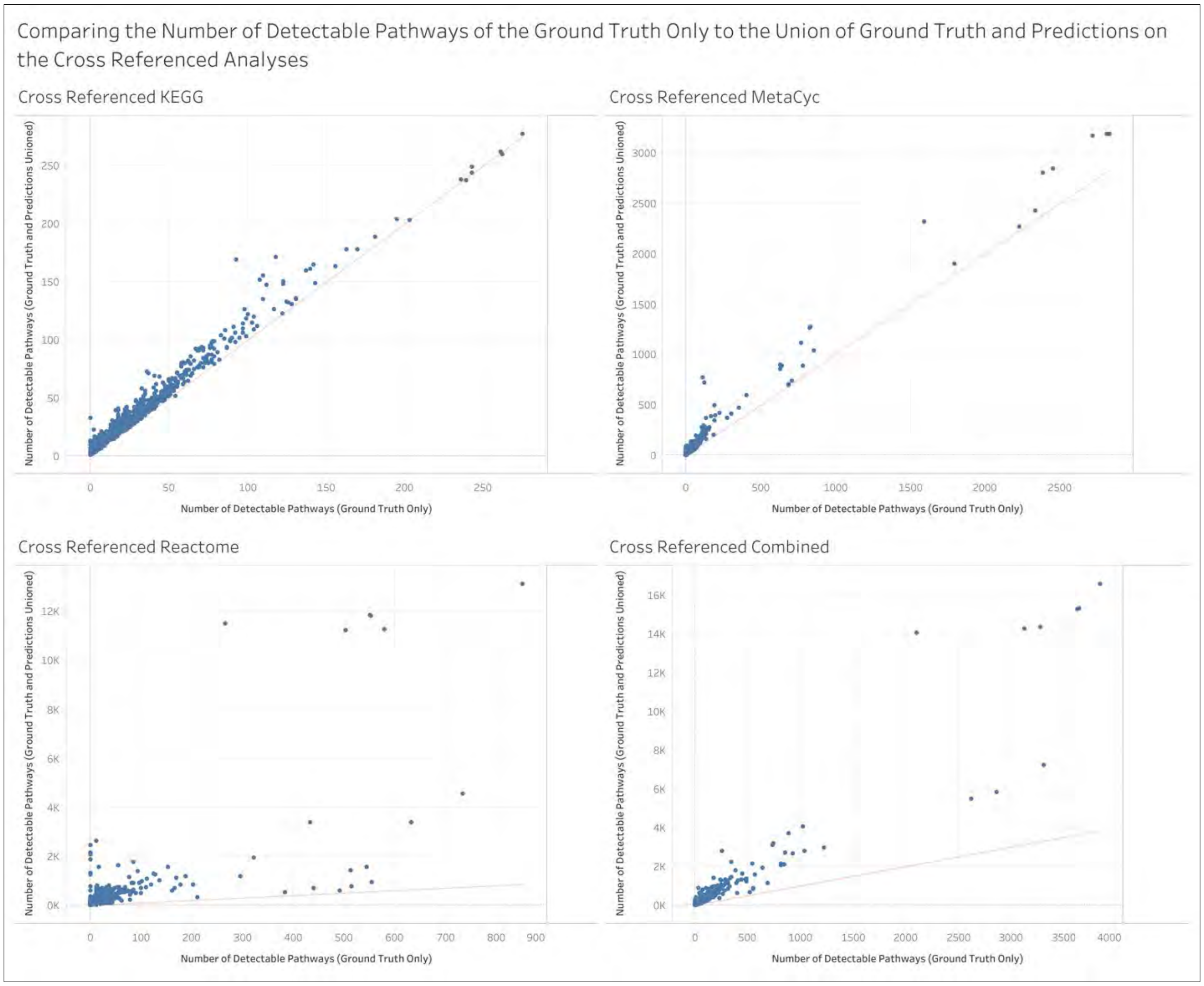

Figure 2 - Per analysis view of the change in the number of detectable pathways between the ground truth and that unioned with predictions for each knowledgebase using cross-referenced MW datasets.

bioRxiv preprint doi: https://doi.org/10.1101/2025.11.18.689105; this version posted November 19, 2025. The copyright holder for this preprint (which was not certified by peer review) is the author/funder, who has granted bioRxiv a license to display the preprint in perpetuity. It is made available under aCC-BY 4.0 International license.

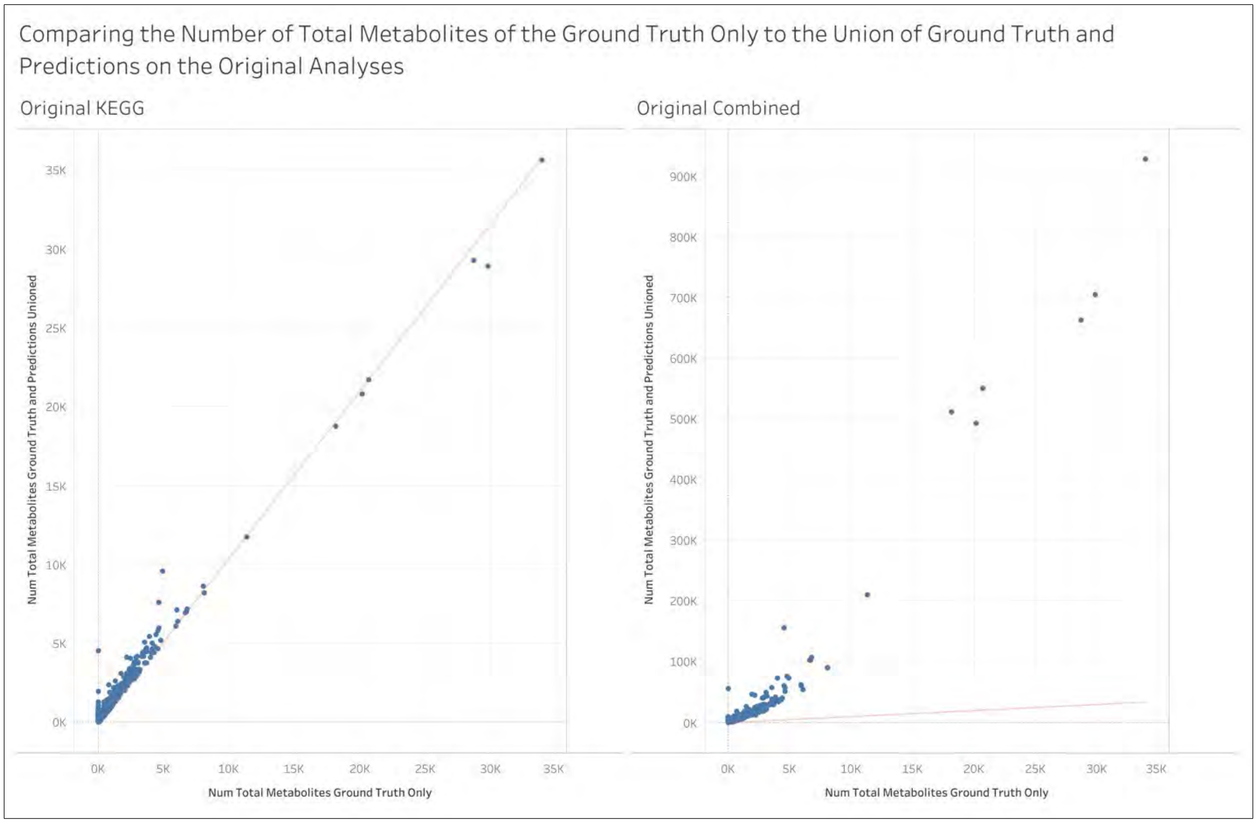

Figure 3 - Per analysis view of the change in the number of detectable pathways between the ground truth and that unioned with predictions for each knowledgebase using the original MW datasets. MetaCyc and Reactome are not shown here since there were not any metabolite IDs available for these in the original datasets.

bioRxiv preprint doi: https://doi.org/10.1101/2025.11.18.689105; this version posted November 19, 2025. The copyright holder for this preprint (which was not certified by peer review) is the author/funder, who has granted bioRxiv a license to display the preprint in perpetuity. It is made available under aCC-BY 4.0 International license.

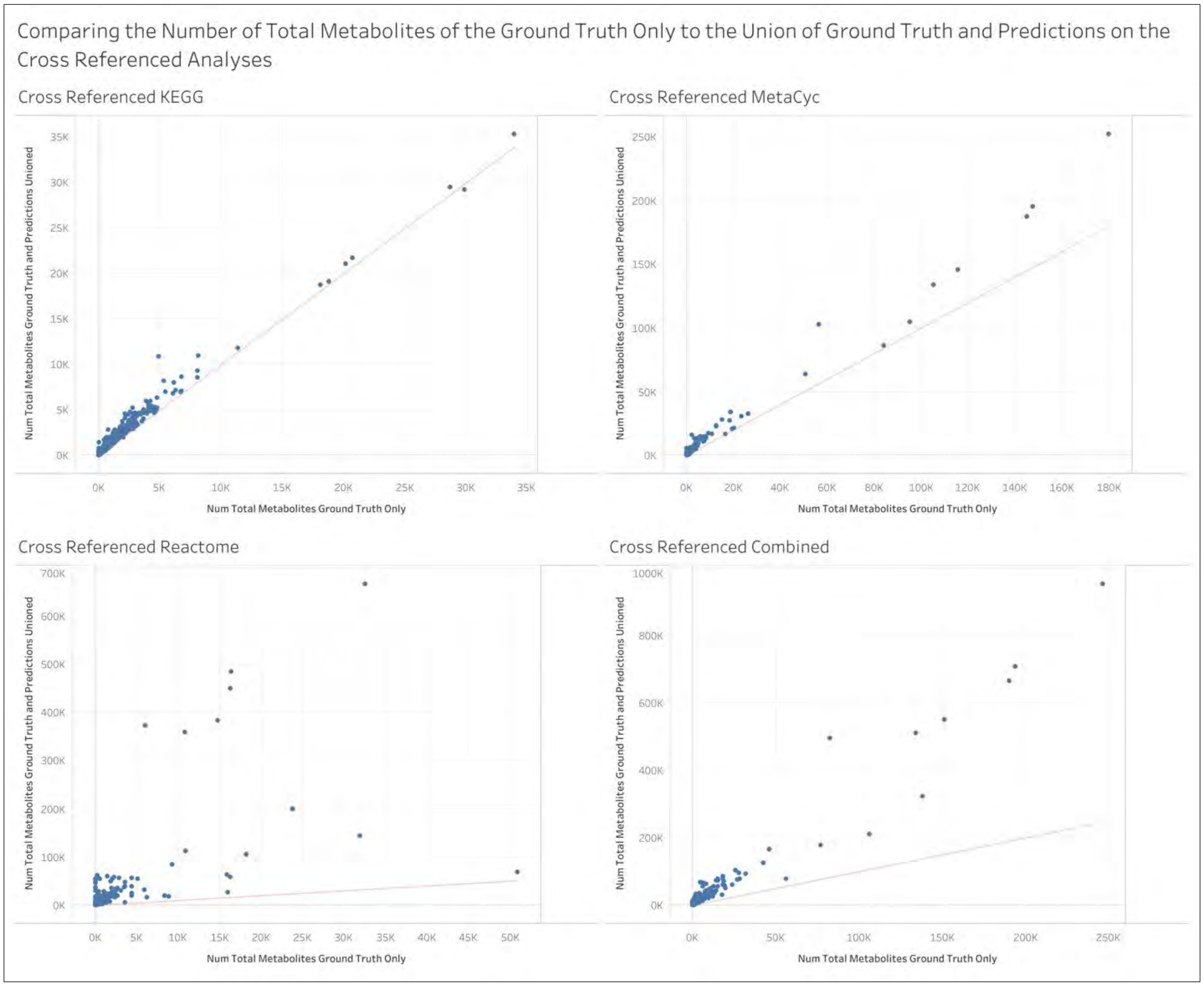

Figure 4 - Per analysis view of the change in the number of metabolite-pathway annotations between the ground truth and that unioned with predictions for each knowledgebase using cross-referenced MW datasets.

bioRxiv preprint doi: https://doi.org/10.1101/2025.11.18.689105; this version posted November 19, 2025. The copyright holder for this preprint (which was not certified by peer review) is the author/funder, who has granted bioRxiv a license to display the preprint in perpetuity. It is made available under aCC-BY 4.0 International license.

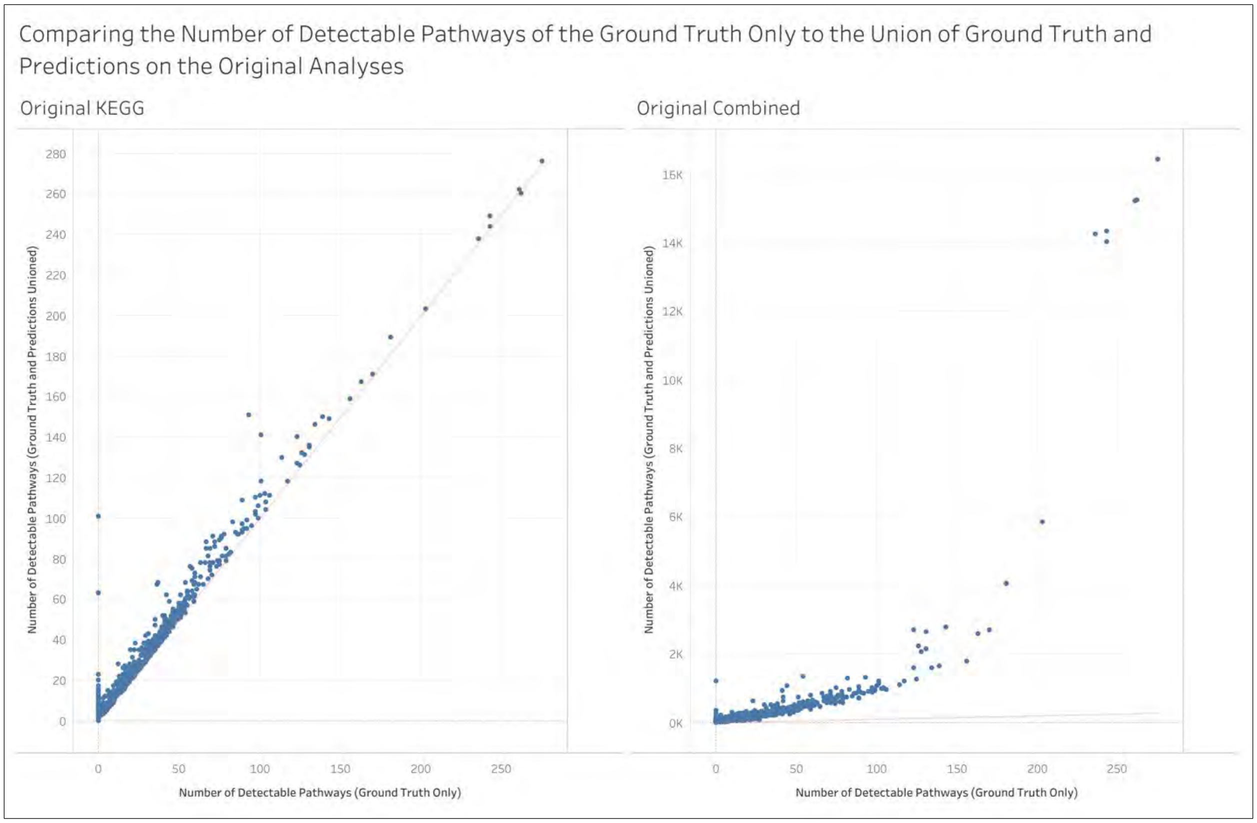

Figure 5 - Per analysis view of the change in the number of metabolite-pathway annotations between the ground truth and that unioned with predictions for each knowledgebase using the original MW datasets. MetaCyc and Reactome are not shown here since there were not any metabolite IDs available for these in the original datasets.

bioRxiv preprint doi: https://doi.org/10.1101/2025.11.18.689105; this version posted November 19, 2025. The copyright holder for this preprint (which was not certified by peer review) is the author/funder, who has granted bioRxiv a license to display the preprint in perpetuity. It is made available under aCC-BY 4.0 International license.

Post-GSEA

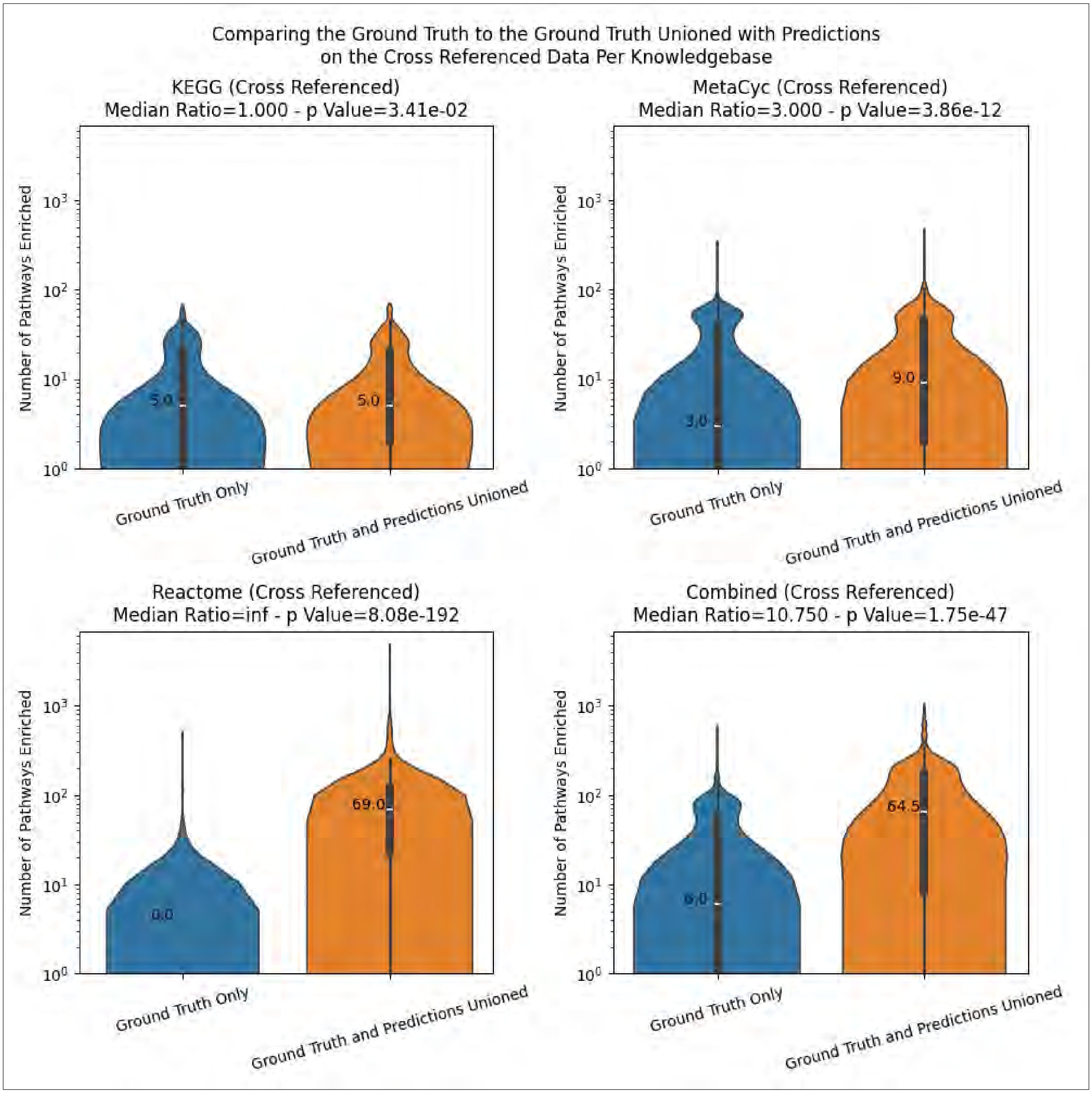

Figure 6 -Comparing the distribution across MW analyses of the number of enriched pathways between the ground truth pathway annotations and those unioned with the predicted annotations for the cross referenced MW datasets for each knowledgebase.

bioRxiv preprint doi: https://doi.org/10.1101/2025.11.18.689105; this version posted November 19, 2025. The copyright holder for this preprint (which was not certified by peer review) is the author/funder, who has granted bioRxiv a license to display the preprint in perpetuity. It is made available under aCC-BY 4.0 International license.

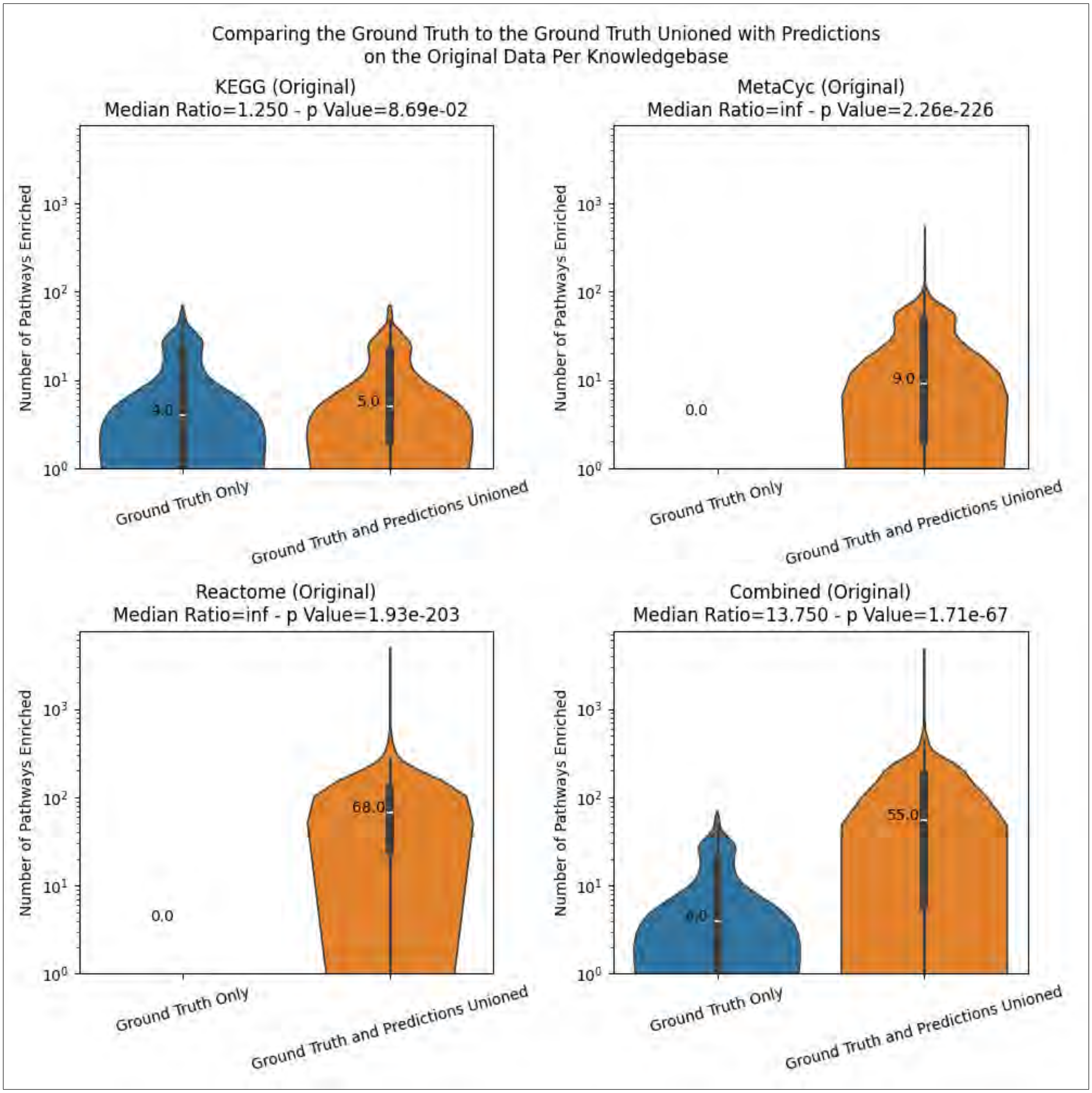

Figure 7 - Comparing the distribution across MW analyses of the number of enriched pathways between the ground truth pathway annotations and those unioned with the predicted annotations for the original MW datasets for each knowledgebase.

. Note that MetaCyc and Reactome had no ground truth annotations available since their metabolite IDs were not available in the original MW datasets.

bioRxiv preprint doi: https://doi.org/10.1101/2025.11.18.689105; this version posted November 19, 2025. The copyright holder for this preprint (which was not certified by peer review) is the author/funder, who has granted bioRxiv a license to display the preprint in perpetuity. It is made available under aCC-BY 4.0 International license.

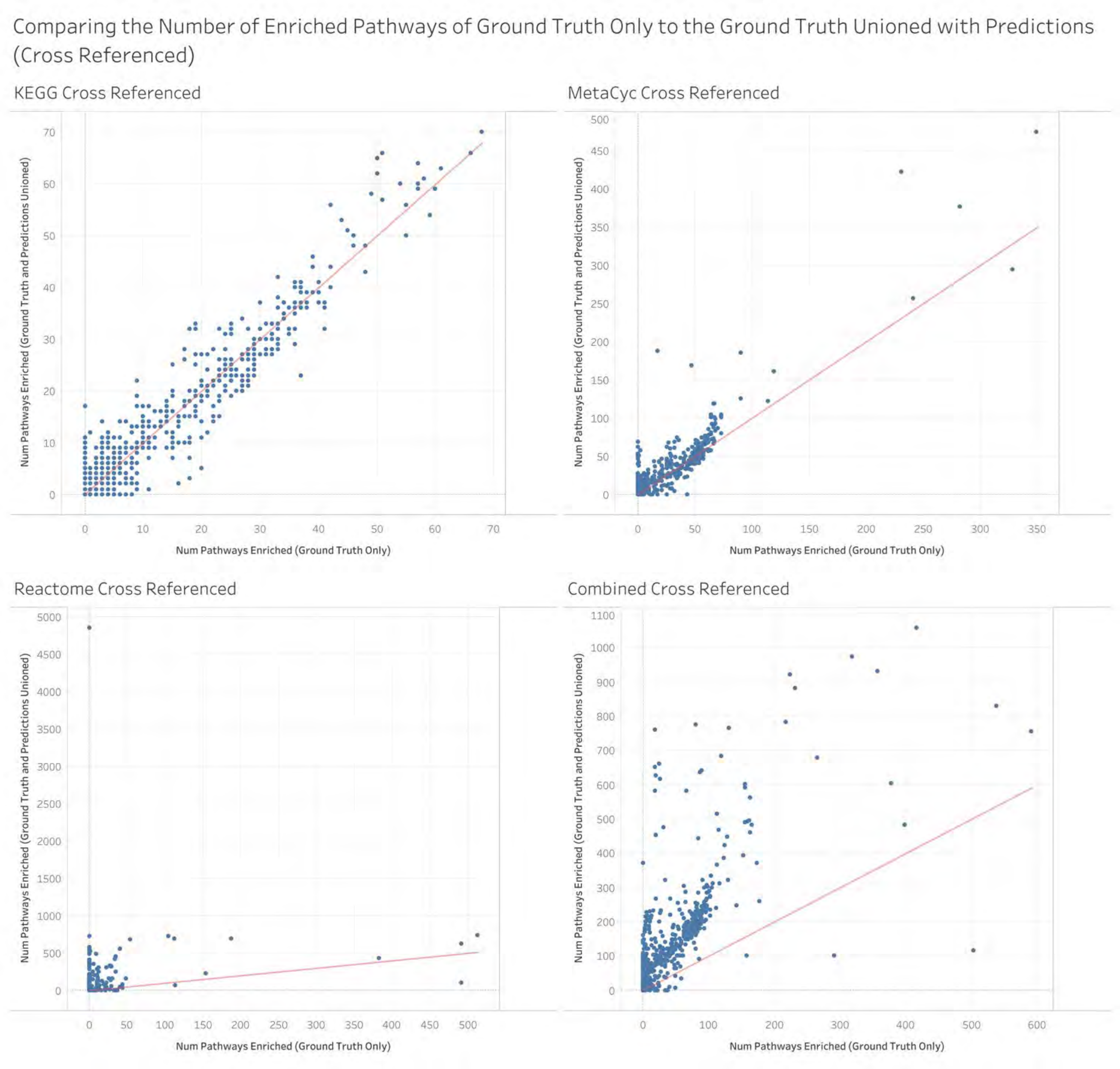

Figure 8 - Per analysis view of the change in the number of enriched pathways between the ground truth pathway annotations and those unioned with the predicted annotations for each knowledgebase on the cross-referenced MW datasets.

bioRxiv preprint doi: https://doi.org/10.1101/2025.11.18.689105; this version posted November 19, 2025. The copyright holder for this preprint (which was not certified by peer review) is the author/funder, who has granted bioRxiv a license to display the preprint in perpetuity. It is made available under aCC-BY 4.0 International license.

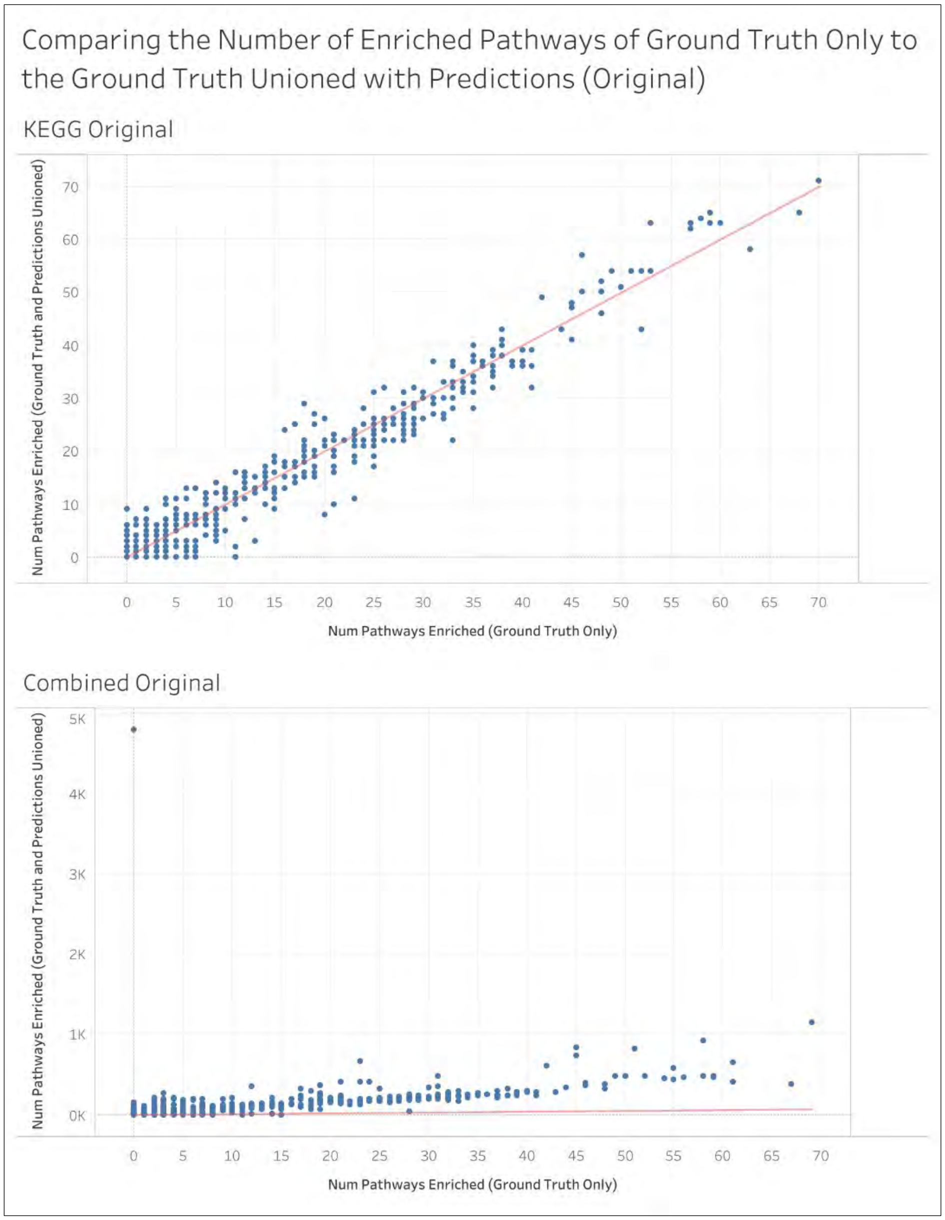

Figure 9 - Per analysis view of the change in the number of enriched pathways between the ground truth pathway annotations and those unioned with the predicted annotations for each knowledgebase on the original MW datasets. Note that MetaCyc and Reactome are not shown because these had no metabolite IDs available in the original datasets.

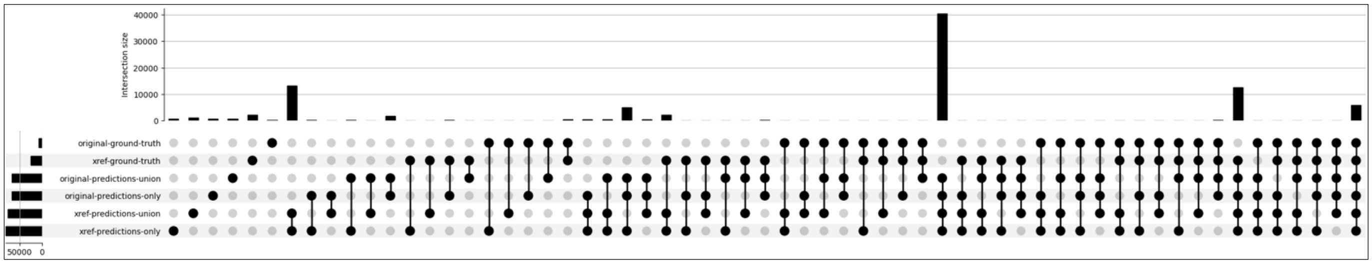

Figure 10 - Represents the information loss and information gain of enriched pathways between ground truth and predictions by showing the overlap of detected pathways.
